# Characterization of immune response induced against catalytic domain of botulinum neurotoxin type E

**DOI:** 10.1038/s41598-020-70929-8

**Published:** 2020-08-18

**Authors:** Priyanka Sonkar, Vinita Chauhan, Ritika Chauhan, Nandita Saxena, Ram Kumar Dhaked

**Affiliations:** 1 Biotechnology Division, Defence Research and Development Establishment, Gwalior, MP 474002 India; 2 Toxicology and Pharmacology Division, Defence Research and Development Establishment, Gwalior, MP 474002 India

**Keywords:** Applied immunology, Cytokines, Immunotherapy, Vaccines

## Abstract

Botulinum neurotoxins (BoNTs) represent a family of bacterial toxins responsible for neuroparalytic disease ‘botulism’ in human and animals. Their potential use as biological weapon led to their classification in category ‘A’ biowarfare agent by Centers for Disease Control and Prevention (CDC), USA. In present study, gene encoding full length catalytic domain of BoNT/E-LC was cloned, expressed and protein was purified using Ni–NTA chromatography. Humoral immune response was confirmed by Ig isotyping and cell-mediated immunity by cytokine profiling and intracellular staining for enumeration of IFN-γ secreting CD4^+^ and CD8^+^ T cells. Increased antibody titer with the predominance of IgG subtype was observed. An interaction between antibodies produced against rBoNT/E-LC was established that showed the specificity against BoNT/E in SPR assay. Animal protection with rBoNT/E-LC was conferred through both humoral and cellular immune responses. These findings were supported by cytokine profiling and flow cytometric analysis. Splenocytes stimulated with rBoNT/E-LC showed a 3.27 and 2.8 times increase in the IFN-γ secreting CD4^+^ and CD8^+^ T cells, respectively; in immunized group (P < 0.05). Protection against BoNT/E challenge tended to relate with increase in the percentage of rBoNT/E-LC specific IL-2 in the splenocytes supernatant (P = 0.034) and with IFN-γ-producing CD4^+^ T cell responses (P = 0.045). We have immunologically evaluated catalytically active rBoNT/E-LC. Our results provide valuable investigational report for immunoprophylactic role of catalytic domain of BoNT/E.

## Introduction

*Clostridium botulinum* is Gram-positive anaerobic, spore-forming bacteria that produce botulinum neurotoxins (BoNTs). Botulinum neurotoxins are considered as the most toxic substances known to humankind^1^ and the causative agent of botulism^2^. BoNTs are also classified by the Centers for Disease Control (CDC) as one of the highest-risk threat agents for bioterrorism (“Class A agents”)^3^. Botulism is characterized by flaccid paralysis induced by blockade of acetylcholine release at neuromuscular junctions that, if not treated, can be fatal^4^. Traditionally, based on their antigenic properties, BoNTs are classified into 7 distinct serotypes (A–G)^5^. Recently a new serotype was reported as BoNT-H^6^, it has since been disproven as a novel serotype, collective data now accurately described it as FA chimeric *i.e.* BoNT/F5A or BoNT/HA rather than a novel new serotype^7,8^. All BoNT serotypes act through similar mechanisms on their target neuronal cells. BoNTs are synthesized as an inactive single polypeptide chain of ~ 150 kDa. It is activated after post-translational cleavage in di-chain consisting of a ~ 100 kDa heavy chain (HC) and a ~ 50 kDa light chain (LC) held together via single disulfide bond^9^. Structurally, these BoNTs are composed of three functional domains of ~ 50 kDa each; receptor-binding domain (HC_C_), translocation domain (HC_N_), and catalytic domain (LC). Intoxication of BoNTs is a multistep process that initiates with the binding of HC_C_ on the presynaptic cell surface followed by internalization of LC mediated by HC_N_ through receptor-mediated endocytosis. Inside the nerve terminal, the LC, which is a zinc-dependent metalloprotease, cleaves one of the three soluble *N*-ethylmaleimide-sensitive factor attachment protein receptors (SNAREs), thereby blocking synaptic vesicle fusion thus release of acetylcholine^10–12^. BoNT serotypes have a unique cleavage site on its target SNARE protein. BoNT/E serotype, which is associated with 25% of human botulism cases, cleaves the 25 kDa synaptosomal-associated protein (SNAP-25) and blocks the neurotransmission relatively faster than other serotypes; possibly due to its uniqueness of domain organization^13,14^.

Till date no effective prophylactic or therapeutic treatment is available for BoNTs intoxication. Subsequently, there is an utmost desire to develop countermeasures against BoNTs. Various medical countermeasures have been developed or undergoing research to prevent botulism. The current treatment for botulism relies upon the passive administrative of antibodies and supportive care. Two passive therapies, heptavalent botulinum antitoxin as investigational new drug (HBAT-IND) that contains equine-derived antibodies against BoNT/A to G for the treatment of adults available by CDC^15^. To treat infant botulism, human-derived immune globulins, BabyBIG is being supplied by the California Department of Public Health (CDPH)^16,17^.

These post-exposure antitoxin therapies have several drawbacks owing to their side effects due to hypersensitivity and are effective towards the extracellular toxin but are unable to neutralize internalized toxin^16^. In addition, there are issues regarding storage and quantity required for mass application^18–20^. Formalin-inactivated penta-serotype-BoNT/A-E (PBT) toxoid vaccine has been used to vaccinate people in the USA to military personals and at high-risk workers^21–23^. In 2011, CDC discontinued the use of PBT vaccine due to its allergic effects^18,24^. In some countries like Japan, the immunogenic efficacy of toxoid treatment was proved against BoNT/E^21^. Prevention can also be achieved by vaccination that generates neutralizing antibodies against botulinum neurotoxin. These toxoids based vaccine made from purified BoNTs. However, mass application necessitates the large-scale production of BoNTs, that require high containment facility and direct handling of toxins which imposes risk for workers. So, to overcome all these problems, continuous efforts being made toward the development of vaccines based on DNA and recombinant proteins that exhibit lesser side effects^25^.

The latest advances in recombinant DNA technology and recombinant protein purification techniques have simplified the production and assessments of complex novel proteins. Many of the BoNTs have been engineered in an effort to produce novel classes of therapeutic molecules^26^. These new generation recombinant vaccines would eliminate the need of specialized manufacture facility and could alleviate the problems related to toxoid vaccine. Some laboratories are involved in the development of recombinant protein vaccines using holotoxins, HCs and LCs. There have been several reports available elaborating the protective potential of holotoxin based vaccines against BoNTs^25,27,28^. Smith and co-workers reported an inactive BoNT/A1 holotoxin protein that provide greater protective potential upon 1,000 LD_50_ toxin challenge^29^. Recently, Webb et al. demonstrated the consistent potencies of trivalent formulation of ciBoNT/C1, /E1 and /F1 (triCEF) against both monovalent and polyvalent toxin challenges found effective as an adjuvanted vaccine at 4–8 °C for up to 2 years^30^.

Indeed, most of the studied targets are HC based which, by directly blocking the binding activity of toxin, consistently show satisfactory protection levels^14,31^. However, efforts have been made to develop vaccine against BoNT/E-LC where, Christine et al., reported development of humanized recombinant BoNT/E-LC antibody displaying neutralization capacity of 5× MLD of BoNT/E at micromolar concentrations^32^. These reports that account for providing protection-using LCs are supported by two reasons; (1) LCs evokes protective antibodies, and (2) produce antibodies that possess the counterintuitive property of blocking toxin action at nerve endings. These combined findings form a rational basis for determining how anti-light chain antibodies including cytokines neutralize botulinum neurotoxins. This in turn could provide essential information for deciding whether the light chains or any related polypeptide should be viewed as an authentic vaccine candidate.

Therefore, this study aimed for the development of effective prophylaxis against botulism. We have focused our study on LC of BoNT/E and examined its immunoprophylactic potential. Accordingly, we have purified nontoxic functionally active recombinant light chain i.e. rBoNT/E-LC used for immunization and production of antibodies. Followed by titer measurement and assessing of protective efficacy their affinity was determined through surface plasmon response (SPR). To the best of our knowledge, this is the first report assessing the humoral and cell-mediated immune responses after rBoNT/E-LC immunization. The cleavage activity assessed by endopeptidase assay using in-house produced rSNAP-25 (substrate of BoNT/E). We have confirmed its humoral response by Ig isotyping and cell-mediated response by cytokine profiling (IFN-γ, TNF-α, IL-2, IL-4, IL-10) and intracellular staining for enumeration of IFN-γ secreting CD4^+^ and CD8^+^ T cells. This rBoNT/E-LC protein induced protection when challenged with 2× MLD of BoNT/E, however no protection was seen at 5× and 10× MLDs.

## Results

### Expression and purification of rBoNT/E-LC

**Figure 1:**
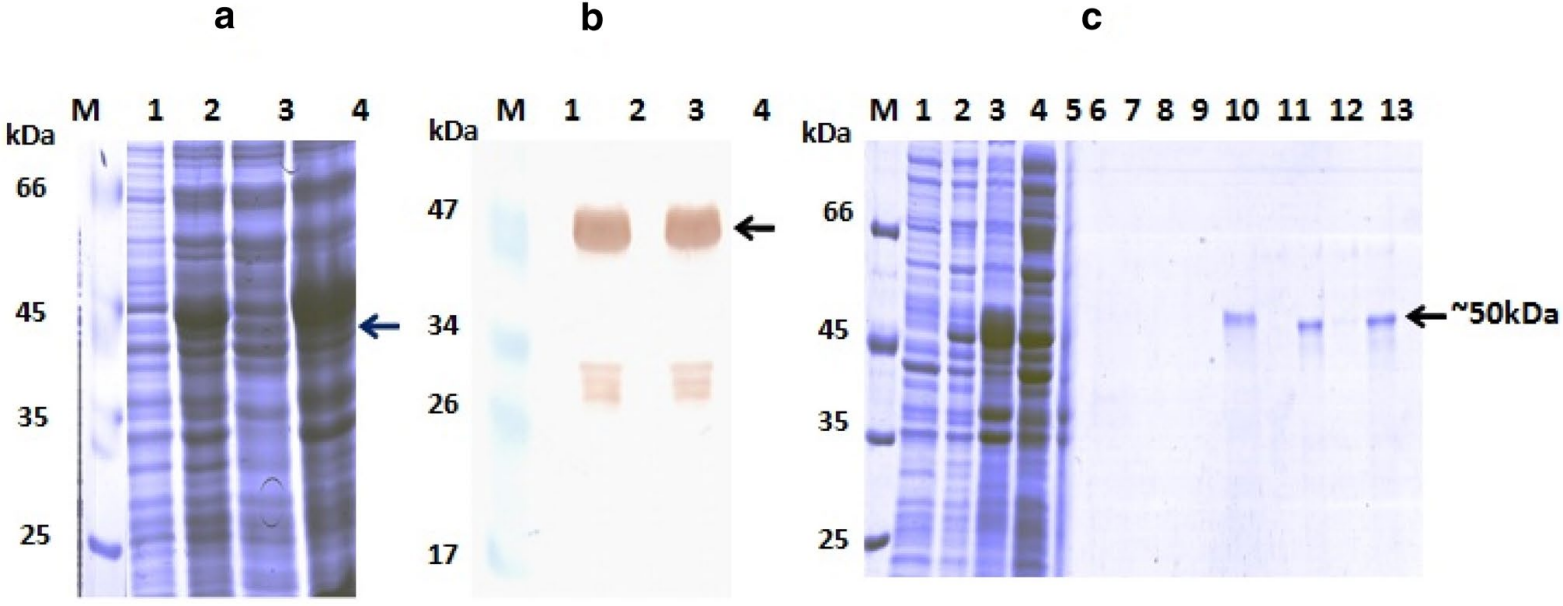
Expression, western blot and purification profile of rBoNT/E-LC (**A**) SDS-PAGE profile of rBoNT/E-LC, (**B**) Western blot profile of rBoNT/E-LC probed with anti His HRP-labelled antibody: lane M: Molecular weight marker; lane 1: uninduced pellet; lane 2: induced pellet; lane 3: supernatant after sonication; lane 4: pellet after sonication. (**C**) Purification profile of rBoNT/E-LC Lane M: Molecular weight marker; lane 1: uninduced pellet; lane 2: induced pellet; lane 3: supernatant after sonication; lane 4: pellet after sonication; lane 5: flow through; lane 6–7: washes; lane 8–12: protein elutes, and lane 13: 1 M imidazole.

The pQE30-UA-E-LC construct was transformed and expressed in the SG13009 *E. coli* cells. The protein was expressed optimally in LB medium when induced at ∼ 0.6 (OD_600_) with 1 mM IPTG at 37 °C. The expression of rBoNT/E-LC was found in the form of inclusion bodies at 37 °C due to overexpression of protein. Various optimization studies were carried out with respect to different media, temperature and IPTG concentrations for obtaining protein in native conditions however, expression was not observed in soluble form. Purified inclusion bodies were solubilized in 6 M GuHCl and after binding to Ni–NTA the effort was proceeded for on-column renaturation by washing with reducing concentration of GuHCl. The protein in native form was eluted with ascending concentrations of imidazole this suggested the proper folding of protein. The expression of His-tag recombinant protein of ~ 50 kDa was confirmed by western blot using anti-his antibody as shown in Fig. 1A, B and characterized by MALDI (Supplementary Fig S1a,b). SDS–PAGE analysis of the eluents revealed the presence of major band of recombinant protein in 250 mM imidazole eluted fractions with an approximate yield of 10 mg protein/L (Fig. 1C). This purified protein was unstable and aggregated during storage and displayed self-catalysis upon long-term storage at 4 °C. To counter these problems 10% glycerol^33,34^ was added in the elution buffer for prevention of autocatalytic activity and aggregation for long-term storage and further applications i.e. immunization of animals and other studies.

### In vitro cleavage activity of purified rBoNT/E-LC on r-SNAP-25

**Figure 2:**
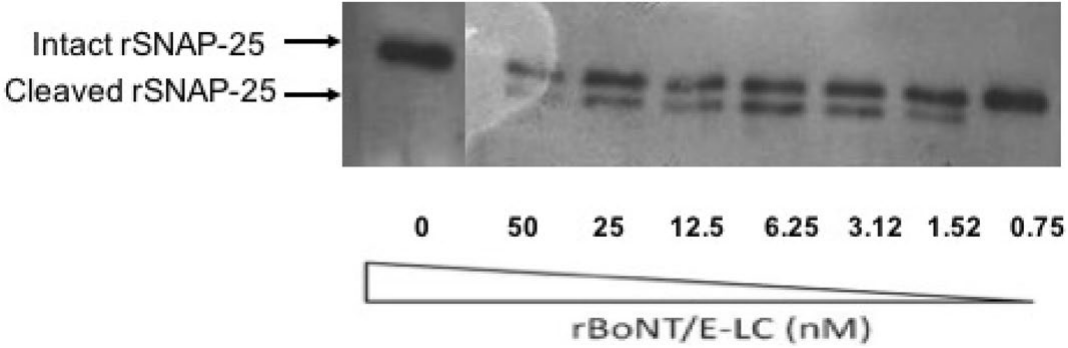
Endopeptidase assay for biological activity of rBoNT/E-LC on rSNAP-25. This assay was performed with fixed concentration of rSNAP-25 (2.5 mM) and incubated with serially diluted concentration of rBoNT/E-LC at 37 °C. Samples were withdrawn after 15 min of incubation, reaction was stopped by adding sample lysis buffer. The immunoblot assay depicts the extent of rSNAP-25 cleavage in rSNAP-25 alone (control). The cleaved rSNAP-25 was analysed by densitometry. The graph illustrates the cleavage activity of purified protein was observed even at the lowest concentration of light chain. Lane 1: rSNAP-25, lane 2–7: rSNAP-25 reaction proceeded with rBoNT/E-LC serially diluted from 100 nM.

**Figure 3:**
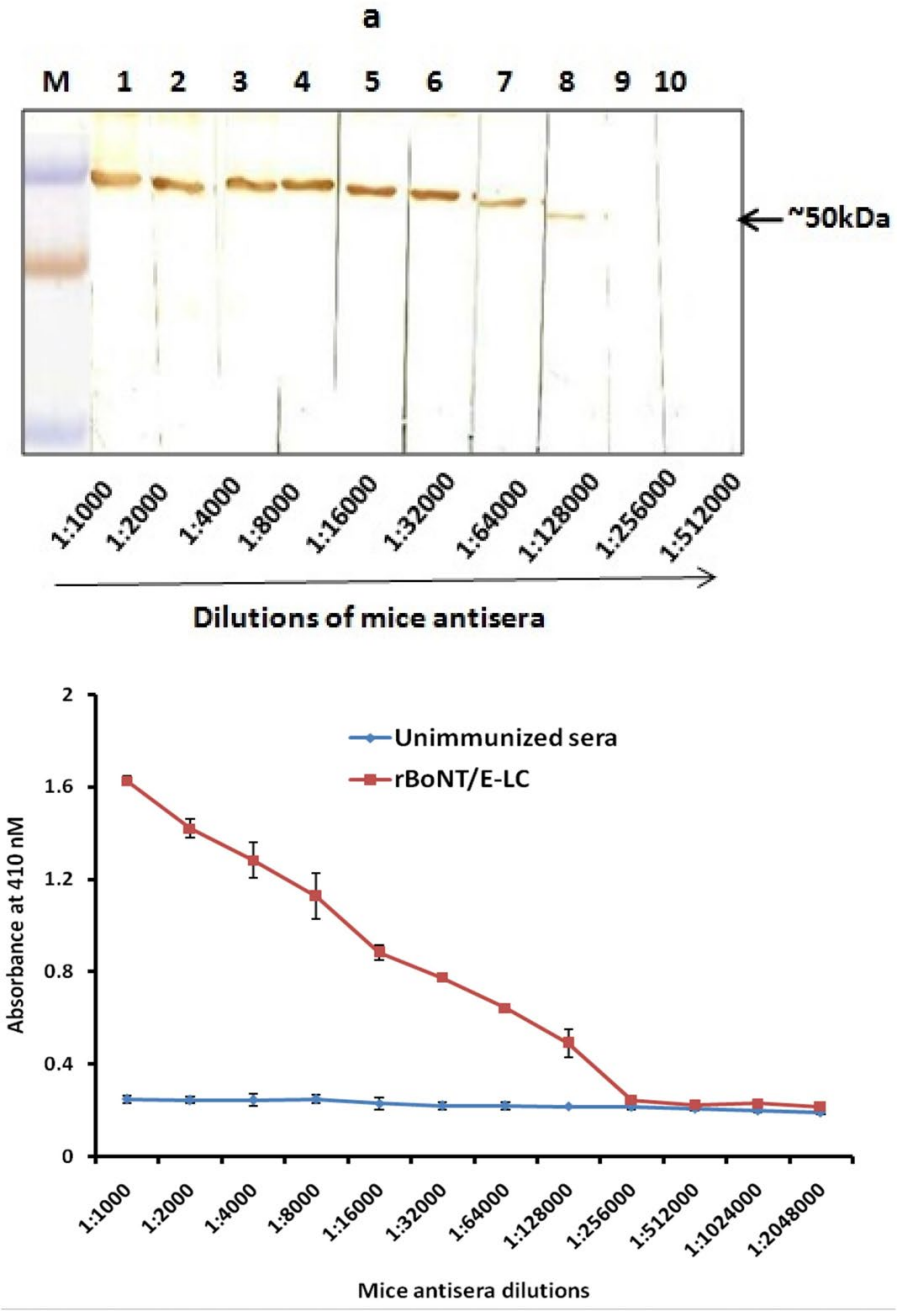
(**A**). Western blot showing the dilution series of antisera raised in mice immunized with rBoNT/E-LC. Lane 1: molecular weight marker indicated to the left; lane 2–11: mice antisera serially diluted from 1:1,000 to 1:512,000 (**B**). Titration curves showing indirect ELISA of mice antiserum that was serially diluted (1:1,000 to 1:2,048,000) and tested against 500 ng/mL of purified rBoNT/E-LC. Data are presented as the mean absorbance ± SD from triplicate wells. Antibody titre was detected upto dilution of 1:128,000 in western blot and 1:256,000 in ELISA, respectively.

The enzymatic cleavage activity of purified rBoNT/E-LC on rSNAP-25 was confirmed through endopeptidase assay. The cleavage activity of rBoNT/E-LC on rSNAP-25 was takes place at position R^180^–I^181^. The biological activity of purified rBoNT/E-LC was determined using optimized concentration of substrate rSNAP-25 of 2.5 µM and serially diluted rBoNT/E-LC. This assay displays the cleavage activity of rBoNT/E-LC at low concentration of 6.25 nM in a dose dependent manner (Fig. 2). The activity of rBoNT/E-LC initiates within 2 min and complete cleavage was visualized in 50 min with 12 nM of rBoNT/E-LC as shown in Fig. 3. The cleavage activity of purified LC on rSNAP-25 suggested significant enzymatic activity and enables their detection. The proteolytic activity was determined by the change in the apparent mobility of SNAP-25 the major product from 25 to ~ 22.75 kDa.

### Induction of anti rBoNT/E-LC antibody

Post immunization of animals with purified rBoNT/E-LC, production of polyclonal antibodies was monitored after each booster and final bleeding was done at 40th day post immunization (dpi). The antibodies titer estimated from western blot was found to be 1:128,000 of serum dilution as depicted in Fig. 3A. Further antibodies titer was determined using indirect ELISA and it was observed up to 1:256,000 illustrated in Fig. 3B. The cut off value for the ELISA was calculated as the mean OD_410_ (± 2 SD) from values of the control group, which was calculated to be 0.24. Further to check whether sera was reactive to other serotypes, western blot was done against light chains of BoNT/A, B and F serotypes and no cross-reactivity was observed that confirmed the specificity of produced antisera (data not shown).

### Evaluation of binding affinity of antibodies with rBoNT/E-LC by SPR

**Figure 4:**
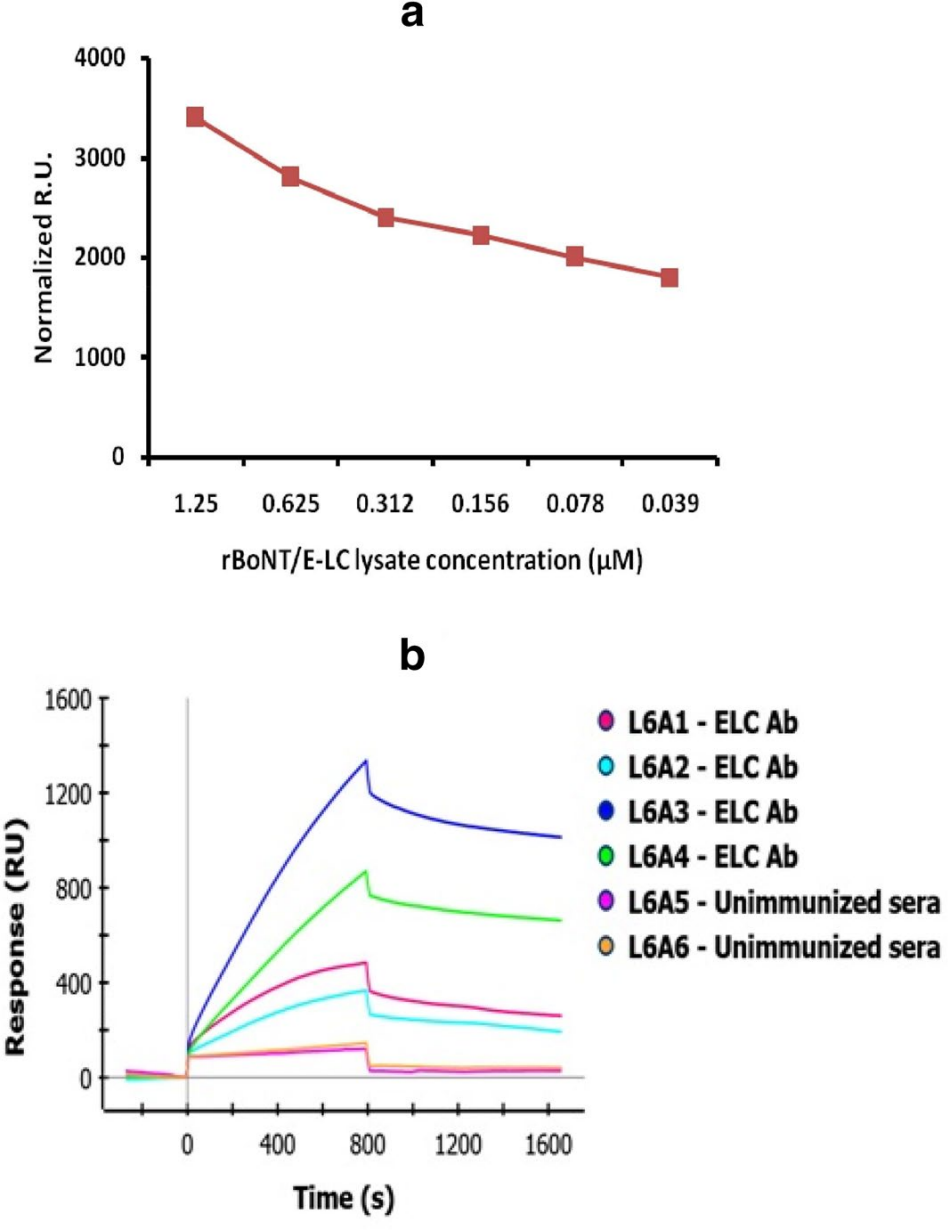
The affinity of antibodies was determined by SPR, His tagged rBoNT/E-LC lysate immobilized on HTG chip: (**A**). The plot here illustrates the lysate binding on the HTG chip in 6 different concentration ranging from 1.25–0.039 μg. (**B**). Sensorgrams obtained for the binding of mice antibodies to immobilized rBoNT/E-LC using ProteOn XPR36 system. The mice antibodies were injected at four different concentrations (shown in different colours) ranging from (7.2–0.0072 μM) and negative control in duplicates. The high RU signifies the greater binding affinity of mice antibodies with rBoNT/E-LC as depicted in graph while unimmunized sera (pink, brown color) showing the fastest dissociation and low RU.

To evaluate the binding affinity between rBoNT/E-LC and antibodies, SPR was used which is a very powerful tool that estimates interaction affinity in label free reaction. Different concentrations of His-tag rBoNT/E-LC from bacterial cell lysate were immobilized on HTG chip. An increase in resonance unit was detected linearly with the increasing concentration of rBoNT/E-LC lysate. The binding of lysate onto HTG chip initiates even at the lowest concentration of rBoNT/E-LC (0.39 µg). The sensorgrams represent the binding of varied concentrations of antigen on HTG chip (Supplementary Fig. S2). Mice antibodies were used as analyte for the interaction with immobilized rBoNT/E-LC. Different concentrations of antibodies were added (7.2, 0.72, 0.072 and 0.0072 µM). The highest concentration of antibodies displayed the ~ 1,500 RU value showing strong binding affinity with rBoNT/E-LC. SPR based analysis of antigen–antibody interaction also confirmed the specificity of raised antiserum (Fig. 4A,B). The unimmunized sera which was used as negative control displayed the ~ 80 RU which showed no significant binding affinity with rBoNT/E-LC.

### Humoral immune response to rBoNT/E-LC

#### Immunoglobulin isotyping

**Figure 5:**
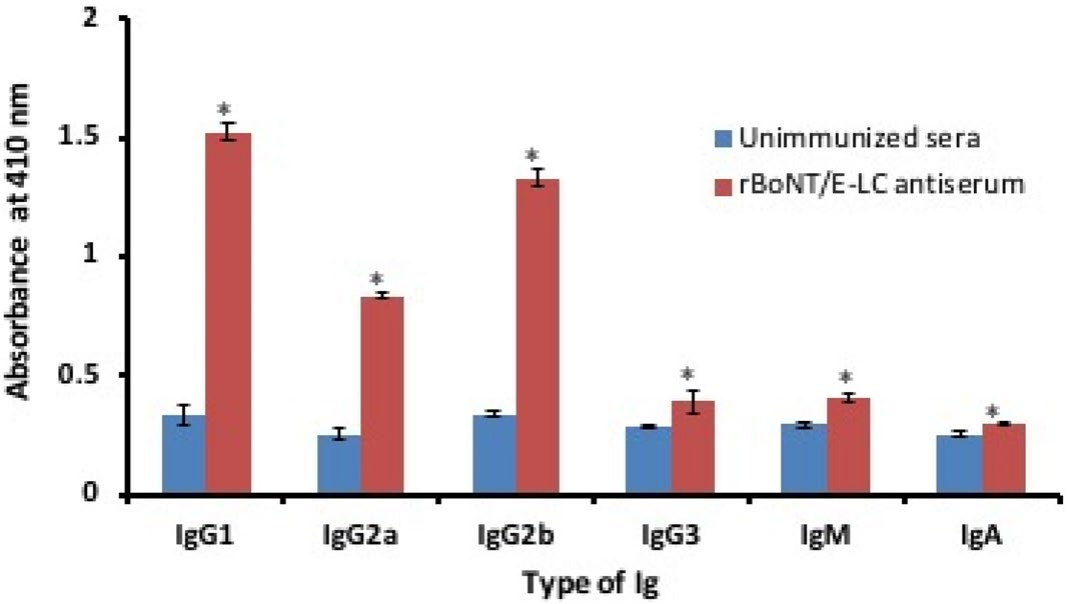
Antibody isotyping: Sera were collected 2 weeks after the last immunization and 1:2,000 dilutions were used. There is significant increase in titre of IgG subtypes in this order IgG1 > IgG2b > IgG2a and insignificant increase observed in IgM followed by IgG3 and IgA. The results are presented as the mean absorbance at 410 nm for each IgG isotype. *p < 0.05 represents statistically significant differences between the immunized and unimmunized groups.

To characterize immune response induced in the immunized mice, the distribution of isotypes of immunoglobulin (Ig) was measured individually in the mice sera from all groups at 2 weeks after the final immunization. The level of specific anti rBoNT/E-LC antibodies in the sera from immunized and control groups was evaluated using ELISA (Fig. 5). Significant levels of Ig specific to rBoNT/E-LC were detected after the last dose of immunization. In contrast, the levels of IgG antibodies in the unimmunized control groups did not statistically increase. As illustrated in Fig. 5, the increase in the levels of IgG1 and IgG2b in the immunized groups (*P < 0.05) were significantly higher than those in the control group.

### Mouse splenocytes cytokine profiling

To determine whether rBoNT/E-LC immunization induces the Th1 or Th2 cytokine response, rBoNT/E-LC Ag-stimulated culture supernatants of splenocytes were obtained from immunized mice two weeks after the final immunization. The levels of IL-2, IL-4, IL-10, IFN-γ, and TNF-α were measured by ELISA. As illustrated in Table 1, the levels of IL-2 in spleen cell cultures from immunized mice were significantly higher (*P < 0.05) as compared to control groups suggesting the proliferation of T-cells activation. In case of IFN-γ expression level, massive increase (fivefold higher) was noticed in immunized groups. A significant difference (*P < 0.05) was observed in immunized group as compared to control. However, the level of IL-4 and IL-10 was statistically not significant than control groups. The slight elevation was also observed in the expression level of TNF-α as compared to control. Elevated levels of IL-2, and IFN-γ suggest induction of type1 helper cell-mediated immune response.

**Table 1:** Cytokines expression levels in different animal groups.

Sr. no	Treatment groups	IL-2 (pg/ml)	IL-4 (pg/ml)	IL-10 (pg/ml)	IFN-γ (pg/ml)	TNF-α (pg/ml)
1	Control	4.48 ± 1.6	34.6 ± 2.2	43.78 ± 2.6	124.3 ± 4.6	50.69 ± 2.15
2	Immunized	15.83 ± 3*	29.4 ± 1.7	31.41 ± 1.01	706.6 ± 16.07*	120.8 ± 4.8*

Significant difference (*P < 0.05).

### Enumeration of CD4^+^ and CD8^+^ T cell population

**Figure 6:**
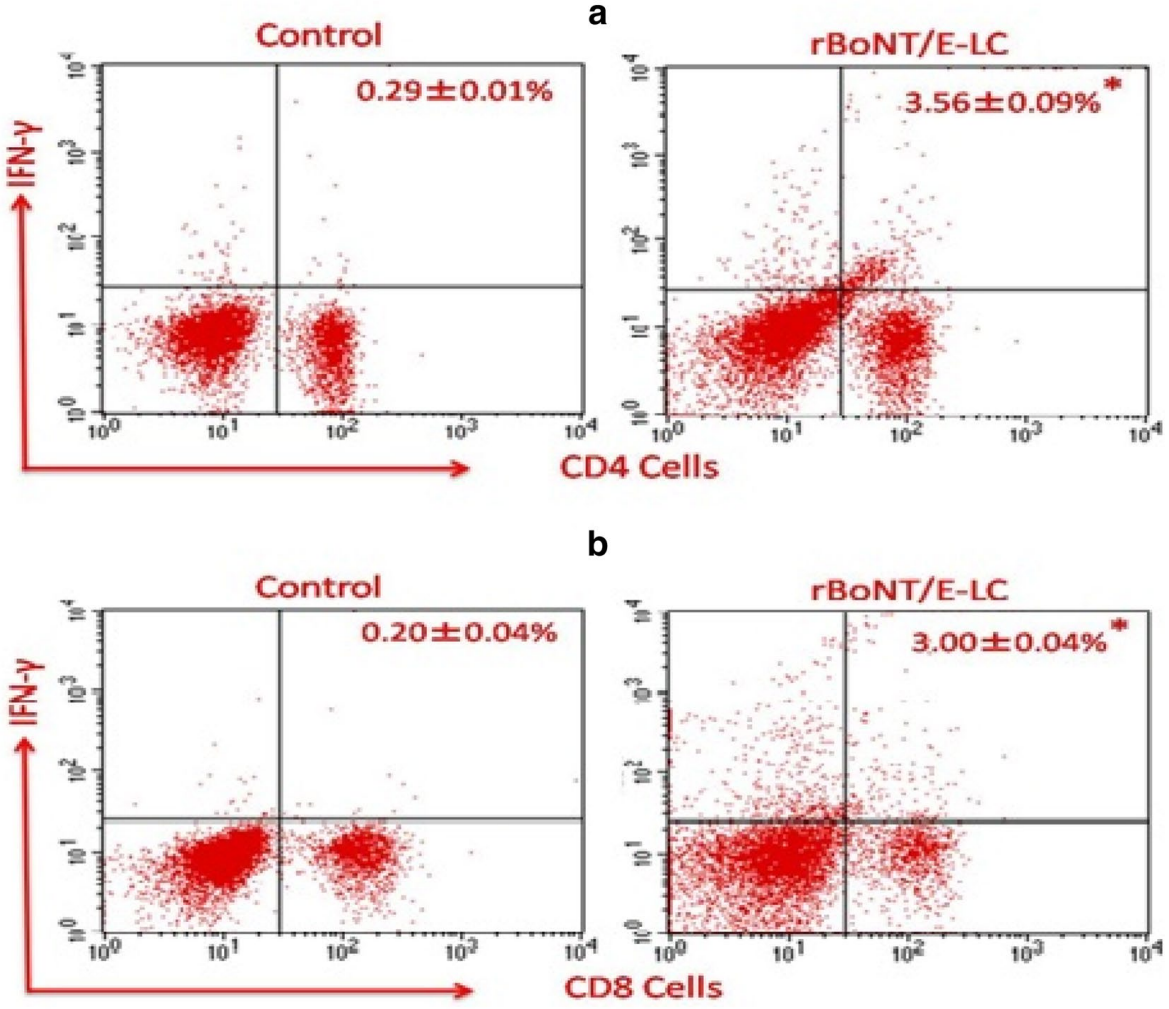
Intracellular cytokine staining of rBoNT/E-LC specific CD4^+^ and CD8^+^ T cells in the splenocytes of immunized mice following stimulation with either negative control or rBoNT/E-LC. Percentage of IFN-γ T cells determined by intracellular staining of stimulated primed T cells. Data are the representative of three independent experiments each in triplicate with standard error: *p < 0.05 compared with unstimulated cells.

The cell-mediated immune response was further elaborated by FACS analysis of the CD4^+^ and CD8^+^ T cell population in isolated splenocytes of immunized and control group animals. This analysis revealed the presence of an increased percentage of the IFN-γ secreting CD4^+^ and CD8^+^ T cells among splenocytes stimulated with rBoNT/E-LC antigen in the immunized group. The population count (%) of IFN-γ secreting CD4^+^ T cells for control and rBoNT/E-LC immunized groups were 0.29 ± 0.01% and 3.56 ± 0.09%, respectively. The population count (%) of IFN-γ secreting CD8^+^ T cells for control and immunized groups were 0.20 ± 0.04% and 3.00 ± 0.04%, respectively. A significant difference was noticed in the IFN-γ secreting CD4^+^ T cells (*P < 0.05) and CD8^+^ T cells (*P < 0.05) to all the immunized groups in comparison to control (Fig. 6). We also observed a remarkable difference between CD4^+^ and CD8^+^ T cells population in rBoNT/E-LC immunized group.

### In vitro neutralization assay

**Figure 7:**
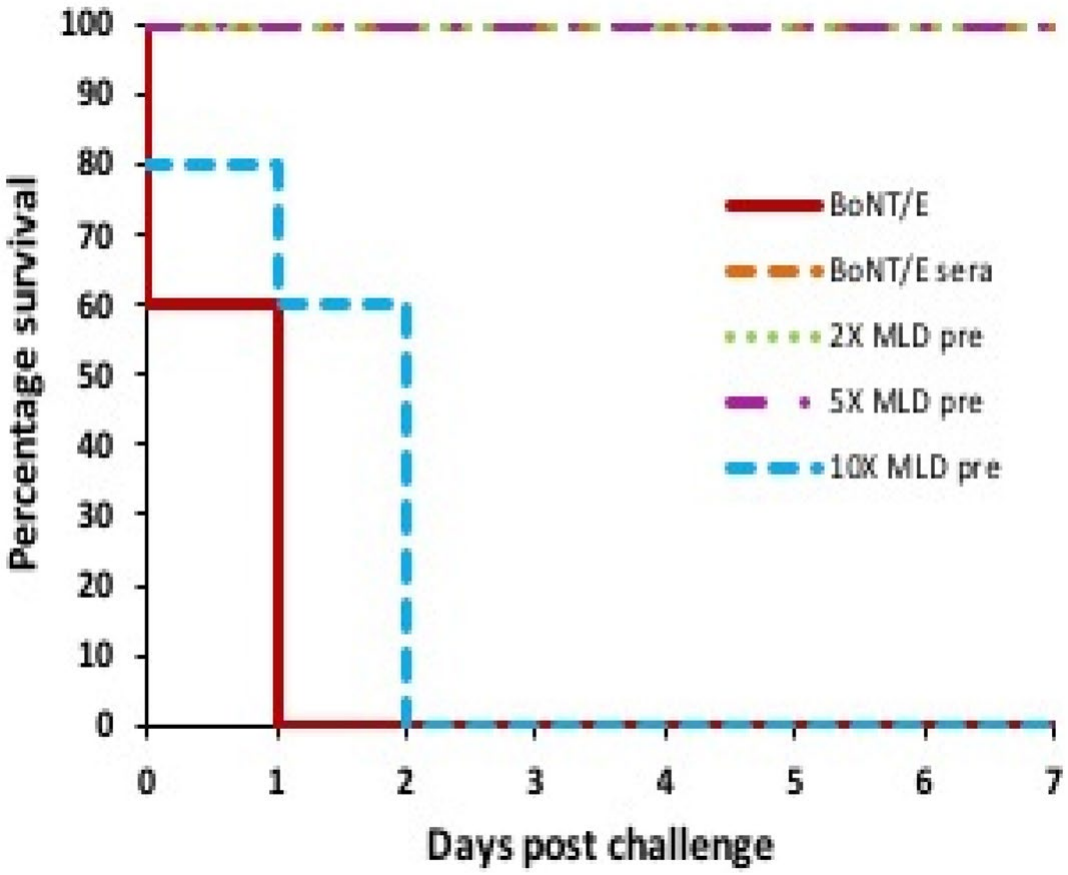
In vitro neutralization study: The naïve BALB/c mice were injected with BoNT/E (brown), BoNT/E sera alone (orange), pre-incubated mixture of vaccinated mice sera: with 2x (green), 5x (purple) and 10x MLDs (blue). This graph illustrates the animals examined for 7 days for their survival.

The confirmation of neutralizing activities of raised antisera against rBoNT/E-LC was determined by in vitro neutralization assay. As depicted in Fig. 7, animal group that received pre-incubated mixture of 2× and 5× MLDs of BoNT/E with antisera were fully protected with no apparent symptoms of toxicity. While no protection was observed in 5th group that was challenged with 10× MLD. The positive control group of mice injected with toxin alone showed symptoms of botulism followed by death within 16 h, whereas no death and symptoms of toxicity was observed in the negative control group of mice that received antisera used in the assay without toxin.

### Evaluation in mice model

**Figure 8:**
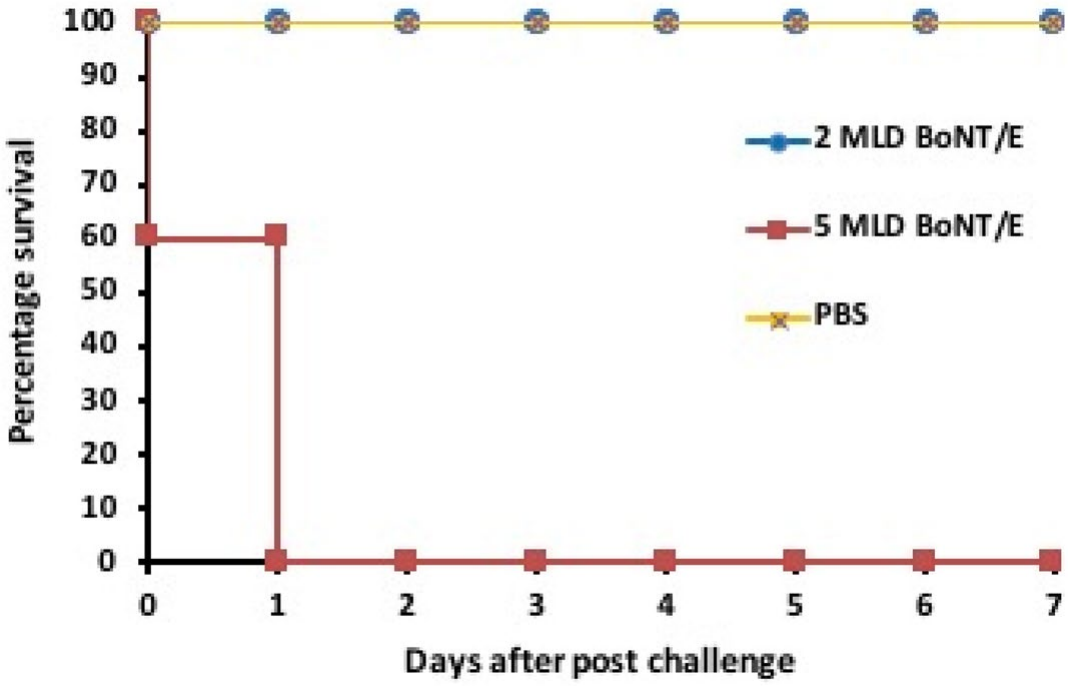
In vivo protection study: the immunized animals were challenged with PBS (control), BoNT/A, B, E and F serotypes. This graph illustrates the immunized animals examined for 7 days after the challenge, immunized mice showed complete protection when injected with 2x MLD of BoNT/E.

Two weeks after the last booster, the immunized mice were challenged with two different doses of BoNT/E and 2× MLD of BoNT/A, B, and F. As shown in graph (Fig. 8), complete protection (100% survival) was observed among immunized mice, challenged with 2x MLD of BoNT/E, while no protection was observed with 5× MLD of BoNT/E. Death was observed among all mice those challenged with 2x MLD of BoNT/A, B, and F (Supplementary Fig. S3) showing no cross protection with other serotypes. The survival of challenged animals was monitored for 7 days.

## Discussion

The toxicity of BoNTs could be known from the fact that they are 100 billion times more toxic than cyanide and million times to sarin^35^. The extreme toxicity (LD_50_ ~ 1 ng/kg), unavailability of antidotes and relative ease of production contributes to their potential as powerful bioweapon agents^36^. In spite of extensive research in the field, no FDA approved treatment is yet available against botulinum neurotoxin intoxication. Current treatment relies upon the use of antitoxins (HBAT-IND and BabyBIG) with several drawbacks. Toxoids elicit some level of protection however; due to its poor potency, annual booster is required. Majority of the patients eventually develop allergic reactions and medical applications restrict mass vaccination^16,37,38^. However, mass application necessitates the large-scale production of BoNTs and requires their purification and toxoid development. Therefore, there is urgent need to develop effective medical countermeasures to combat these complications.

Numerous efforts are being made to develop novel vaccines as effective medical countermeasures targeting holotoxins and different/combinations of domains to substitute the existing toxoid vaccine. Researchers have studied holotoxin that can be exploited as potential vaccine candidate to prevent intoxication. It involves several strategies like introducing point mutations in holotoxin to eliminate catalytic activity^28^. Another successful attempt done by Webb et al., by using inactive holotoxin and their data strongly suggests that the ciBoNT HP may offer a robust immunological response that elicits greater protective immunity, particularly when challenges are performed with dissimilar toxin subtypes^27^. It was assumed that inclusion of all three-protein domains could provide greater diversity of potential neutralizing epitopes against that antibodies generated^29^. The potency of these holotoxin based vaccine was found consistent^30^.

As we know the structure of BoNTs consist of two polypeptide chains that remain joined by di-sulphide bridge until internalized into neurons. Major previous studies towards vaccine development are focused on HC^39,40^ and LC-based vaccine^41,42^, but most reports primarily available for type A and B^43–45^. These recombinant vaccine candidates have been produced in *Escherichia coli*, and other bacteria. The sub-unit vaccines are advantageous as these can be produced in required quantities in low/no containment facility. The LC subunits as a vaccine formulation can offer protection and prove to be advantageous as it is cheaper, efficacious, easily accessible and non-hazardous. The mechanism of protection in animals after immunization with both subunit vaccines is to block the action of toxin.

Previous studies reported the production and purification of BoNT/E-LC, however, did not mention its immunological characterization^46^.

Herein, we purified the rBoNT/E-LC responsible for the cleavage activity of the toxin. The production of functionally active light chain is crucial for biological activity and immunological characterization.

The rBoNT/E-LC was purified using affinity chromatography and confirmed by western blotting. The purified protein rBoNT/E-LC are prone for autocatalysis and was not stable upon storage for longer time. Therefore, the stability of protein was assessed by addition of 10% glycerol in the storage buffer that prevents the aggregation of the protein as reported in previous studies^33,34^. The biologically active ~ 98.5% pure protein was produced. The analysis of functional activity of rBoNT/E-LC was determined by endopeptidase assay using its in-house purified rSNAP-25 substrate. This assay imparted the important and valuable information confirming the proper structural and stability of LC.

We performed elaborate immunological characterization and profiling of the response generated by LC of BoNT/E. The purified rBoNT/E-LC used for the immunization and generation of antibodies in BALB/c mice. We investigated the detailed immunological profiling of rBoNT/E-LC by estimating cytokines and characterizing antibodies from immunized animals.

The titer obtained from ELISA was significantly higher as compared to preimmunized sera (Fig. 3). The affinity of antibodies towards rBoNT/E-LC was determined through label-free SPR assay and observed in significant RU as compare to the control serum. The raised serum was specific to BoNT/E and not cross-reactive with other serotypes, which was confirmed by western blot.

In order to better characterize the induced immune response, IgG1 and IgG2a titers in the sera of immunized mice were analysed by Ig isotyping. The significant increase was observed in the level of IgG1 and IgM subtype after the initial booster dose. Subsequent to final dose, predominant increase was observed in the IgG1 and IgG2b followed by IgG2a. This Ig subtyping study suggested that rBoNT/E-LC immunization involves mostly B lymphocyte activation, thereby, generating Th2 type immune response. Concerning the induction of humoral responses and IgG subtypes, as shown in Fig. 5, immunized group indicated significantly elevated levels of IgG1 and IgG2 in comparison to control group. Interestingly, the levels of IgG3, IgM and IgA were lower than control group. The final response thus, can be concluded as mixed or a balanced immune response of Th1 and Th2 types.

The humoral immunity relies upon production of antibodies by antibody-producing plasma B cells, which effectively neutralize extracellular pathogens and provide protection from bacterial toxin^44,47^. Since, humoral immunity alone is not enough for effective protection; the cell-mediated immunity plays a key role in inducing strong immune response. In an effort to elaborate the comprehensive immunological impact, study was further expanded for estimating cytokines to study cell-mediated response. Splenocytes were isolated from immunized mice and stimulated for 48 h with rBoNT/E-LC protein. We measured different cytokines expression level including IL-2, IL-4, IL-10, IFN-γ and TNF-α in mice splenocytes. We observed two-fold increase in the expression level of IL-2 suggesting the proliferation of T cells. The massive increase in the level of IFN-γ expression as well as inferior level of elevation in pro-inflammatory cytokine TNF-α was also observed as shown in Table 1. The expression level of IL-2 was elevated and mostly induced specific response as detected in immunized mice after final dose immunization. Furthermore, the elevated rBoNT/E-LC-specific IFN-γ and IL-2 cytokine levels than the control group suggests presence of a stronger Th1 response. The cytokine production is the prime step of immune response as it provides the necessary information of any immunologic responses. The protection induced by cell-mediated immune response against intracellular pathogen relies upon the production of a Th1 type of immune response, development of pathogen-derived antigen-specific IFN-γ and TNF-α secreting T cell.

In addition to cytokine profiling, the measurement of T cell activation was further assessed by intracellular cytokine staining (ICS) assay. ICS by flow cytometry permits the analysis of specific T cell responses in outlined cell populations compared to different techniques like ELISpot. The detection of cytokines in appropriate populations of T lymphocytes initially characterized was based on the surface and intracellular expression of CD4^+^ (helper T cells) or CD8^+^ (cytotoxic T cells). The quantification of IFN-γ secreting CD4^+^ and CD8^+^ cells in immunized animals were analysed by flow cytometry. Among immunized mice splenocytes, mainly IFN-γ secreting CD4^+^ cells population was higher than the unstimulated control group and the marked differences were observed between immunized and control group (P* < 0.05). Secretion of IFN-γ is considered a marker of the Th1 cell-mediated immune response. A significant increase in rBoNT/E-LC specific CD4^+^ and CD8^+^ T cell populations was recorded in immunized group. There was strong correlation observed between the intracellular and secreted cytokine when data of ICS and cytokines levels were compared. Our data of cytokine and antibody typing revealed that the LC was immunogenic in animals, eliciting assessable humoral and cell-mediated immune responses to rBoNT/E-LC. The LCs of botulinum neurotoxins are potentially immunogenic and generate protective immune responses and antibodies against BoNTs^34,48^. The exact mechanism of LC antibody in protection against botulism is unclear^42^. However, the possible mechanism for neutralization of toxin has been revealed in a study conducted on cell culture where it was hypothesized that antibodies against LC force BoNTs to return to the cell surface through synaptic vesicle recycling^41^.

The protection at higher doses by antibodies of BoNT/E-LC is still disputed. The in vitro neutralization and in vivo protection assay clearly displayed the efficacy of polyclonal antibodies in preventing intoxication through BoNT/E at lower doses. However, the present study was not able to protect animals at higher doses. In fact, it is necessary to evaluate our LC antigen in combination with HC to elucidate its protective potential on higher doses. In addition, results that are more precise might be obtained by combining heavy chain to light chain and the same study will be done. This study is the first report aiming on BoNT/E immunological characterization.

Results of in vivo study suggest that rBoNT/E-LC alone cannot act as vaccine candidate in spite inducing the production of IFN-γ, TNF-α producing T cells and IL-2 as estimated in culture supernatants stimulated with rBoNT/E-LC. The incorporation of anti-LC antibodies in passive antitoxin therapy or LC subunit along with another HC based vaccine formulation might prove advantageous and could enhance its efficacy. It can contribute in intracellular neutralization and toxin-antibody complex clearance that will provide additional layer of protection. These findings shed light on the interesting aspect of inability of rBoNT/E-LC in presenting viable vaccine candidature, however it generates profound immune response.

## Conclusion

There is utmost need to develop effective medical countermeasures against BoNTs. For this development we produced biologically active recombinant BoNT/E-LC that generated protective immune response. The high affinity specific antibodies were produced and immunologically characterized. The protein is soluble, nontoxic, catalytically and biologically active, and is stable for a prolonged period. This study is the first report of complete immunological profiling induced by LC of BoNT/E.

Extensive immunological characterization and profiling performed displays stimulation of immune response by rBoNT/E-LC. However, rBoNT/E-LC alone does not have the requisite ability to elicit significant protection in mice. Immunization with rBoNT/E-LC provided protection in animals challenged with lower doses but failed to protect at 5× MLD. But the contribution of the individual domains in the development of vaccines is important. In the last two decades after the recent advances in recombinant vaccine, significant research has been done in heavy chain based vaccine. However, only a few studies are available in case of light chain based vaccine specially type E. Reports available on antibodies against both the LC and HC and this information could potentially serve as a means for the elucidation of immunoprophylactic potential of both the subunits. This kind of study has not been reported yet. In this report the production of rBoNT/E LC to study the immunological response to the catalytic domain of the toxin, and in upcoming studies as a reagent to be used in high-throughput assays to screen for potential LC inhibitors. In conclusion, our results indicated successful cloning, production and immunological characterization of LC. Investigation of the immunogenicity and immunoprotectivity of these fragment could extend our understanding about their implication for immunoprophylaxis and treatment of botulism.

## Materials and methods

### Ethical statement and biosafety

This study was conducted in according to Committee for the Purpose of Control and Supervision of Experiments on Animals (CPCSEA) and was approved by the Institutional Animal Ethics Committee (IAEC) of Defence Research and Development Establishment, Gwalior, India (Permit number: AH/05010/CPCSEA/2018 and protocol no. BT-37/57/RKD). Experiments conducted at the DRDE, Gwalior, approved by the Institutional Animal Ethics Committee. All the toxin challenge experiments were conducted in high containment facility, biosafety level-3 at DRDE, Gwalior, India.

### Biochemicals and reagents

Trypticase peptone yeast extract glucose (TPYG) and Luria Bertani (LB) medium were purchased from Difco Laboratories, USA. *Escherichia coli* SG13009, pQE30-UA vector, Ni–NTA resin, plasmid DNA purification kit (27,106), and PCR Kit were procured from Qiagen, USA. Pre-stained protein markers, unstained protein molecular weight marker, (SM0431), DNA ladder (SM0313) were obtained from Fermentas, USA. Primers were synthesized from Microsynth, Switzerland. Cell culture media components, monoclonal anti-polyhistidine antibody raised in mouse (H1029), HRP conjugate AP163P, Anti-His HRP antibody, mouse monoclonal antibody isotyping reagents (ISO2) and chicken anti-goat Ig antibody, 3, 3′-diaminobenzidine (DAB), and other required chemicals were purchased from Sigma Aldrich, USA. Cytokine ELISA kit & flowcytometry reagents were obtained from BD Biosciences, USA.

### Cloning of BoNT/E-LC gene

Genomic DNA was isolated from anaerobically overnight grown *C. botulinum* type E in TPYG culture media at 37 °C. PCR amplification of full length BoNT/E-LC gene was carried out using forward primer 5′-GGA TCC ATG CTG TAT ATG CCA AAA ATT-3′ and reverse primer 5′-GAA TTC TTA TGT CGA CAT ACA TAT TGA TTT CCT TAT GCC-3′. Optimum temperature settings were 95 °C (5 min) initial denaturation; 30 cycles of 95 °C denaturation, 55 °C annealing and 72 °C extension (1 min each); followed by 10 min incubation at 72 °C. Gel extracted purified PCR product of ~ 1,296 bps was cloned into pQE30-UA vector and transformed into *Escherichia coli* host strain SG13009.

### Expression and purification of rBoNT/E-LC

The colony PCR and sequence confirmed clones of rBoNT/E-LC were inoculated into 5 mL of LB medium containing ampicillin (100 mg/mL) and kanamycin (30 mg/mL) and grown at 37 °C. Cultures at logarithmic phase (OD_600_ 0.6) were induced with 1 mM isopropylthiogalactoside (IPTG) and 3 h post-induction cells were harvested by centrifugation (6,000 × *g*). To check the expression profile, supernatant and pellet obtained from induced cell lysate were analyzed on 10% SDS–PAGE. After several washes with wash buffer (50 mM sodium phosphate buffer, 300 mM NaCl, 1% triton, beta-mercaptoethanol; pH 8.0) purified inclusion bodies were dissolved in denaturing agent guanidine hydrochloride (6 M GuHCl). The recombinant proteins carrying histidine tag at C-terminus were purified by immobilized metal affinity chromatography (IMAC) using Ni–NTA column. On column renaturation was adopted for proper refolding of protein^47^. The concentration of the purified protein was estimated by BCA kit (Sigma, USA) and analyzed through SDS-PAGE followed by western blot. The purified protein fraction was dialyzed against 10% glycerol containing PBS (pH 7.4) before use.

#### Biological activity of rBoNT/E- LC

The activity of rBoNT/E-LC was determined by in vitro endopeptidase assay using His-tag rSNAP-25 substrate. The cleavage activity was performed in dose dependent manner in a final reaction volume of 20 µL. This assay was performed by incubating the fixed concentration rSNAP-25 of 5.0 nM with varying concentration of rBoNT/E-LC in reaction buffer (25 mM Tris, 100 mM NaCl, 19.2 mM glycine, 100 μg/mL BSA, 0.1 mM dithiothreitol (DTT), 10 μM ZnCl2, pH 7.5) and incubated for 30 min at 37 °C and the reaction was stopped by adding the 4 × dye sample lysis buffer and boiled at 100 °C for 5 min. The reactions were resolved by 13% SDS-PAGE followed by western blot using monoclonal antibody rabbit anti-SNAP-25 (Sigma Aldrich, USA) in a dilution of 1:5,000, as primary antibody. The goat anti rabbit HRP conjugate antibody was used as secondary antibody in a dilution of 1:20,000. The reactions were detected by chemiluminescence using an ECL western blot kit (Biological industry, Israel) as per manufacturer's instructions. Film was exposed for 15 s before development. The percentage of cleaved substrate was measured by densitometry. The recombinant SNAP-25 vector was transformed into BL21DE3 host cells and rSNAP-25 was purified as abovementioned method for light chain under native conditions and used as substrate.

### Immunological characterization

#### Animal care and immunization of animals

The BALB/c mice (20–25 g, 5–6 weeks old male) were used for immunization, protection and challenge studies. All the animals were housed at temperature 25 ± 2 °C and 12:12 light: dark period at animal house facility of Biotechnology Division, DRDE, Gwalior, India. Animals were fed on the standard pellet diet and water ad libitum.

All animals were randomly allocated to two groups and assigned as I and II: group I were used for immunization with rBoNT/E-LC and further subdivided to A and B: group-IA: involves 30 mice used for evaluation of IgG antibody response and protection studies against toxin challenge. Group-IB involved 10 mice used for cytokine profiling and estimation of CD4^+^ and CD8^+^ T cells while group-II consisted of 10 unimmunized healthy mice and used as control group for standard control toxicity of BoNTs and other studies.

The purified rBoNT/E-LC was used for immunization of BALB/c mice. The 10 μg of antigen was dissolved in PBS emulsified with an equal volume of complete Freund's adjuvant (FCA) (Sigma-Aldrich, USA) for priming and incomplete Freund's adjuvant (FICA) (Sigma-Aldrich, USA) for subsequent booster and injections were administered in each animal through intramuscular (i.m.) route (100 μL/injection). We have taken all precautionary measure to avoid leakage of injection and injury to the mouse. The priming was done on day 0 followed by three boosters on day 7, 14 and 21. Blood was collected after first and each booster doses by retro-orbital sinus bleeding on day 0, 28 and 40. For further studies, all the mice were also bled for sera prior to immunization. Serum was prepared as reported elsewhere^34^. After completion of immunization, sera and splenocytes were collected for assessing of antibody titer, Ig isotypes and different cytokines.

### Evaluation of antibody titers by western blot

Antiserum obtained from immunized mice was screened for anti rBoNT/E-LC polyclonal antibodies by immunoblotting. Purified rBoNT/E-LC (10 µg) was separated on SDS-PAGE (10%) using prep comb and processed for western blotting. After transfer of protein to nitrocellulose membrane (N/C) it was blocked in 3% skimmed milk powder prepared in PBS and cut into strips. Primary incubation in serum samples raised from immunization was two-fold serially diluted beginning from 1:1,000 to 1:512,000 for 1 h at 37 °C. The membrane was washed thrice with PBST (0.05% Tween-20) and twice with PBS for 5 min each. The N/C membrane was incubated for 1 h at 37 °C with rabbit anti-mouse IgG HRP conjugated secondary antibody (1:2,000). Post washing as above, the presence of protein was visualized by 5 min incubation at room temperature with 3,3′-diaminobenzidine (DAB) and hydrogen peroxide.

### Antibody titer determination by ELISA

Titer of anti-rBoNT/E-LC antibodies were detected in the hyper-immune sera qualitative indirect ELISA assay, 96-well microtiter plates (Nunc-Immuno Plate, Denmark) were coated with 500 ng/mL rBoNT/E-LC antigen in 1X coating buffer (0.05 M carbonate-bicarbonate buffer, pH 9.6), for overnight at 4 °C. The plates were then blocked for 2 h at 37 °C with 300 µL per well of 3% bovine serum albumin (BSA) in PBST. Thereafter, plates were washed thrice with PBST and once with PBS. The final bleed antiserum was two-fold serially diluted in 1% BSA beginning from 1:1,000 to 1:4,096,000 and added to the wells in triplicate. The naïve mouse serum was used as negative control. Plates were incubated for 1 h at 37 °C followed by the washing steps. The anti-mouse HRP (1:5,000) antibody was used as secondary antibody and incubated for 1 h at 37 °C. After washing, the plates were incubated with ABTS [2,2′-azino-bis (3-ethylbenzothiazoline-6-sulphonic acid)] and hydrogen peroxide (100 µL/well) for 15 min at 37 °C. The ELISA reader (BioTek Instruments, USA) was used to measure the absorbance of appeared colour at 410 nm.

### SPR based biomolecular interaction

For the present immunological characterization study, ProteOn XPR36 (BioRad Laboratories, USA) SPR system with six parallel flowing channels was used to uniformly immobilize six ligands on the sensor chip gold surface. SPR is an optical technique used for detecting biomolecular interactions. The analysis of interaction can be interpreted by monitoring change in the position of reflected intensity or resonance, which is expressed in the form of response unit (RU). This change in incident angle is recorded in the form of sensorgrams. HTG sensor chip was used for immobilization of His tagged recombinant protein, which was activated with 10 mM NiSO_4_, preceded by conditioning as described previously^49^. The interaction initiated with the immobilization of the ligand (rBoNT/E-LC) in six different concentrations (1.25, 0.625, 0.312, 0.156, 0.078 and 0.039 μg) on the HTG sensor chip surface. After ligand immobilization, the ProteOn XPR36 fluidic system automatically rotates horizontally so that six different analyte can flow simultaneously over all the immobilized concentrations of ligand. Immobilization is transient by means of capturing through vertically oriented multi-channel modules (MCM). Once the binding occurs, the optimum concentrations of ligand (0.039 μg) were selected for interaction. The ligand immobilization step was followed by PBST washing. Then, four different concentrations of mice polyclonal antibodies (7.20, 0.720, 0.072 and 0.0072 µM) were injected to the channels by horizontally oriented MCM that result in binding to the immobilized ligand. Unimmunized serum was used as negative control. The association of ligand and analyte interaction was performed for preset amount of time i.e. 800 s. Their interaction produces a change in refractive index at the gold surface, which was analyzed with ProteOn Manager Software 3.1.

### Evaluation of humoral immune response

The rBoNT/E-LC specific serum Ig subtypes antibody level was measured using rBoNT/E-LC and performed by ELISA, the microtiter plate was coated with rBoNT/E-LC (500 ng/mL) in coating buffer (bicarbonate buffer, pH 9.6) by incubating at 4 °C overnight. It was followed by blocking with 3% BSA for 2 h at RT. After removing the blocking solution, washing of plates was done. Anti-rBoNT/E-LC antibodies were added in 1:2,000 dilutions and incubated for 1 h at 37 °C. After washing, goat anti-mouse IgG1, IgG2a, IgG2b, IgG3, IgM and IgA isotype specific antibodies in 1:2000 dilutions were added and incubated for 1 h. After three washes, HRP labeled chicken anti-goat IgG antibody (1: 2,000) was added and incubated for 1 h. Again after washing, colorimetric substrate (ABTS + H_2_O_2_) was added and incubated for 15–20 min in dark and the reaction was stopped by adding 2 N H_2_SO_4_ and absorbance read at 410 nm. The incubations of antibodies and substrates were done at 37 °C.

### Evaluation of cell-mediated immune response

#### Cytokine profiling

For estimation of cytokines, three mice from immunized group were randomly selected and sacrificed and their splenocytes were removed aseptically and resuspended in RPMI-1640 media with 10% FBS. The splenocytes from un-immunized mice group were used as negative controls. The spleen cells were seeded in a 24-well plate at a density of 1 × 10^6^ cells/well. The isolated splenocytes cultures were stimulated with 5 μg/mL of rBoNT/E-LC. These cells were incubated at 37 °C in CO_2_ incubator (5% CO_2_) for 48 h. Supernatants were collected after centrifugation (1,500 × *g*) and stored at – 80 °C until analyzed for cytokines expression. The expression levels of different cytokines i.e. IFN-γ, TNF-α, IL-2, IL-4 and IL-10 were determined by ELISA using BD OptEIA Kits, according to the manufacturer's protocol. The expression of cytokines was calculated using standard curves generated with known concentrations of recombinant cytokines and expressed in picograms per milliliter (pg/mL).

### Estimation of CD4^+^ and CD8^+^ cell secreting IFN-γ by flow cytometry

For estimation of rBoNT/E-LC specific IFN-γ secreting CD4^+^ and CD8^+^ cells, splenocytes were isolated from immunized and un-immunized animals (as described above). The splenocytes were seeded at 1 × 10^6^ cells/well and stimulated with 5 µg/mL of rBoNT/E-LC antigen. The anti-mouse CD28 (10 µl/mL) was used for co-stimulation and Brefeldin A (BFA) (1.0 µg/well) was added to prevent the transport of protein from the Golgi apparatus to the endoplasmic reticulum. After approximately 5 h of incubation with antigen (37 °C and 5% CO_2_), erythrocytes were lysed by adding fluorescence-activated cell sorting (FACS) lysing solution. Splenocytes were then washed with FACS staining buffer. After washing, cells were subjected to surface staining. For intracellular cytokine staining (ICS), cells were stained for 30 min with fluorescein isothiocyanate (FITC) labelled rat anti-mouse monoclonal antibodies (BD Biosciences, USA) to the CD4 and CD8a cell surface markers; fixed and permeabilized with Cytoperm/Cytofix solution. Later, cells were washed and treated with phycoerythrin-conjugated rat anti-mouse IFN-γ antibody for 30 min at RT in dark. Unstimulated specimens were used to measure basal level of cytokine production. Stained cells were washed and acquired in Becton Dickinson, FACS Calibur Flow-Cytometer apparatus. Total 10,000 live events were observed and analyzed using Cell Quest Pro Software (BD BioScience, USA).

### Preparation of toxin

The BoNT/E standard culture was grown under anaerobic conditions using Clostridial broth/TPYG media (Sigma-Aldrich) at 37 °C for 96 h. The toxin was collected by precipitation with 25% ammonium sulphate and isolated by following the method of Chauhan et al. with slight modification^34^. The culture supernatant was collected by centrifugation, activated by trypsinization (200 μg/mL) (Sigma-Aldrich, USA) followed by anti-trypsin treatment (400 μg/mL) (Sigma-Aldrich, USA) then aliquoted, and stored at − 20 °C.

To determine toxicity, BoNT/E was ten-fold serially diluted in phosphate buffer (pH 7.4) and a volume of 100 μL of each dilution was injected i.p. into 2 male BALB/c mice (20–22 g). The mice were observed for 7 days and symptoms were recorded. The mouse lethal dose (MLD) of each toxin is MLD Unit, defined as the amount of toxin injected intraperitoneal challenge (i.p.) into mice resulting in 100% deaths within their respective death time period. The highest dilution resulting 100% death of both mice was subsequently two-fold serially diluted and a volume of 100 μL of each dilution was injected i.p. into four mice. The highest dilution resulting in 100% death of all four mice was selected as containing 1 MLD of BoNT/E. Toxin of A, B and F are purified previously reported^34^ and their MLD values for BoNT/A were; 00.2 pg , BoNT/B; 3 ng , BoNT/E; 6 ng and BoNT/F 14 ng. Standard control for toxicity analysis of all BoNTs serotypes (A, B, E and F) was performed using other 30 unimmunized healthy mice (5 mice/group) with one group of mice used as vehicle control.

### In vitro neutralization assay

The in vitro neutralization potency of raised antisera against BoNT/E was examined by using naïve BALB/c mice. One hundred µl of antisera obtained from the immunized mice were incubated with different doses of BoNT/E for 1 h at 25 °C prior injecting i.p. into naïve mice. In this process, 30 naïve mice were taken and divided into 5 groups (5 mice/group): 1st group assigned as positive control group injected with toxin alone (5 mice), 2nd group as negative control injected with antisera alone, and three doses of toxin (2×, 5× and 10× MLDs) mixed with antisera and this mixture were injected into 3^rd^, 4^th^, and 5th groups, respectively. The challenged mice were observed for 7 days and death or survival was recorded.

### Evaluation of protective potential in mice model

In order to observe the protective efficiency, the immunized mice were challenged i.p. with 2× and 5× MLDs BoNT/E after two weeks of final bleeding via i.p. route. For cross protection study the immunized mice were challenged with 2× MLD of BoNT/A, B and F. All toxins were diluted in phosphate buffer saline (PBS) pH 7.4. Survival of all challenged animals was monitored for 7 days.

### Statistical analysis

Results are presented as the mean ± standard deviation (SD) of 3 independent experiments. All data comparisons were examined for significance by Student’s t-test. Differences were considered statistically significant when P value is lower than 0.05.

## Supplementary information

Supplementary Information.
