# Supplementary material for: Characterization of immune response induced against catalytic domain of botulinum neurotoxin type E
Source: Sci Rep. 2020 Aug 18;10:13932. doi: 10.1038/s41598-020-70929-8 (PMC7434876; doi:10.1038/s41598-020-70929-8)
Supplement: Supplementary file 1 — Supplementary Information. [file 41598_2020_70929_MOESM1_ESM.docx]

**Characterization of immune response induced against catalytic domain of botulinum neurotoxin type E**

**Priyanka Sonkar, Vinita Chauhan, Ritika Chauhan, Nandita Saxena^1^ and Ram Kumar Dhaked^*^**

*Biotechnology Division, 1- Toxicology and Pharmacology Division, Defence Research & Development Establishment, Gwalior, MP-474002, India*

*corresponding author at

Dr. Ram Kumar Dhaked

Biotechnology Division, Defence Research & Development Establishment (DRDE), Jhansi Road, Gwalior 474002, M.P, India

Email address: ramkumardhaked@hotmail.com


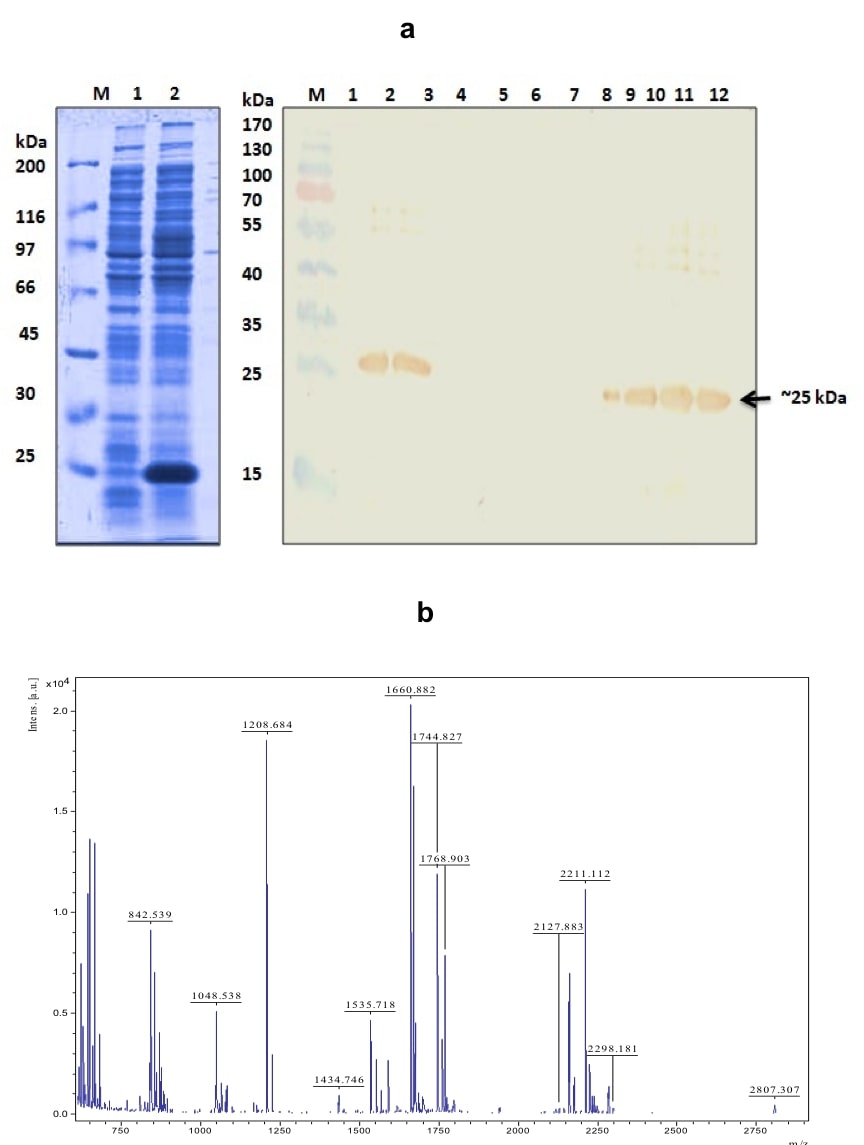


**Figure S1:** Purification and characterization of rSNAP-25 substrate. **(a**) The expression of histidine fusion protein induced culture of rSNAP-25 revealed a presence of ~25 kDa protein band. SDS-PAGE gel showing: Lane: M: unstained marker; 1: uninduced; 2: induced pellet. **(b)** The induced LB culture was purified under native condition using Ni-NTA affinity chromatography. The western blot analysis of purified rSNAP-25 protein using anti-His antibody. Lane: M: prestained marker; 1: uninduced; 2: induced; 3: supernatant after sonication; 4: pellet after sonication; 5: flow through; 6-7: W1-W2: wash fractions; 8-11: E1–E4: eluate fractions; 12: 1 M imidazole. 13 % SDS-PAGE gel showing 25 kDa His tagged protein which was purified under native condition to be used as a substrate to confirm the cleavage activity of rBoNT/E-LC for endopeptidase assay. **(c)** The purified protein band was cut out, digested with trypsin and characterized further with mass spectrometry analysis. MALDI-TOF/MS spectrum of rSNAP-25 protein tryptic digests in reflectron mode using HCCA as matrix. Labelled peaks correspond to the matched peptide *i.e.* 16 peptides were matched with 54% coverage to synaptosomal-associated protein-25 (*Mus musculus*) (NCBI entry: gi|6755588) available in the database.


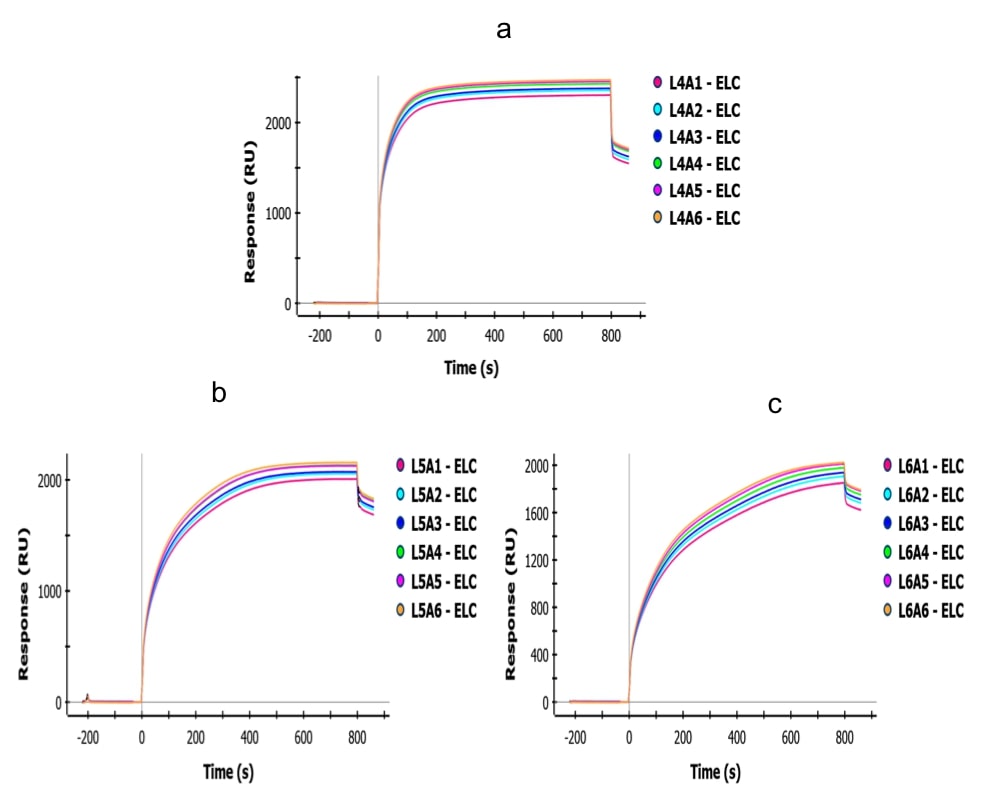


**Figure S2:** Optimization of concentration of rBoNT/E-LC lysate on HTG chip: The sensorgram values as observed in SPR: the immobilization of the ligand (rBoNT/E-LC) in six different concentrations (1.25, 0.625, 0.312, 0.156, 0.078 and 0.039 μg) on the HTG sensor chip surface. Here, sensorgram graph showing the RU of three concentrations (a:0.156; b:0.078; c: 0.039 μg). The optimum concentration of lysate was found to be 0.039 μg and selected for interaction with antibody.


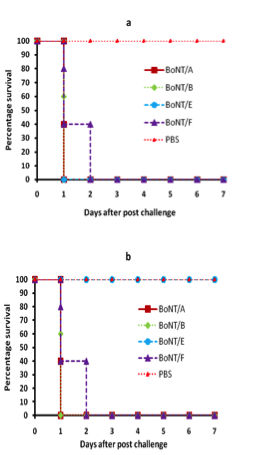


**Figure S3:** *In vivo* protection study: **(a)** The non-immunized BALB/c were administered intraperitoneally with 2X MLD of BoNT/A, B, E & F serotypes and PBS only. The representative graph illustrates the challenged animals were examined for 7 days and their survival and death were recorded. None of the animal group was protected when challenged with BoNT/A, B, E and F. While, in case of those animal group that received PBS alone showed no symptoms of toxicity and no death was observed. **(b)** The immunized BALB/c were injected with PBS (control), 2X MLD of BoNT/A, B, E & F serotypes. This graph illustrates the challenged animals were examined for 7 days and their survival and death were recorded. Death was observed among those animal group when challenged with BoNT/A, B and F. Whereas, all mice were survived that administered with BoNT/E toxin and PBS alone showed complete survival. No symptoms of toxicity were seen among BoNT/E challenged mice.
